# The Anti-Cancer Effect of Polyphenols against Breast Cancer and Cancer Stem Cells: Molecular Mechanisms

**DOI:** 10.3390/nu8090581

**Published:** 2016-09-21

**Authors:** Ahmed Abdal Dayem, Hye Yeon Choi, Gwang-Mo Yang, Kyeongseok Kim, Subbroto Kumar Saha, Ssang-Goo Cho

**Affiliations:** Department of Stem Cell & Regenerative Biotechnology, Incurable Disease Animal Model and Stem Cell Institute (IDASI), Konkuk University, Gwangjin-gu, Seoul 05029, Korea; ahmed_morsy86@yahoo.com (A.A.D.); hyeon.choi24@gmail.com (H.Y.C.); slayersgod@nate.com (G.-M.Y.); proproggs@naver.com (K.K.); subbroto@konkuk.ac.kr (S.K.S.)

**Keywords:** polyphenols, breast cancer, anti-cancer activity, autophagy, apoptosis, cancer stem cells

## Abstract

The high incidence of breast cancer in developed and developing countries, and its correlation to cancer-related deaths, has prompted concerned scientists to discover novel alternatives to deal with this challenge. In this review, we will provide a brief overview of polyphenol structures and classifications, as well as on the carcinogenic process. The biology of breast cancer cells will also be discussed. The molecular mechanisms involved in the anti-cancer activities of numerous polyphenols, against a wide range of breast cancer cells, in vitro and in vivo, will be explained in detail. The interplay between autophagy and apoptosis in the anti-cancer activity of polyphenols will also be highlighted. In addition, the potential of polyphenols to target cancer stem cells (CSCs) via various mechanisms will be explained. Recently, the use of natural products as chemotherapeutics and chemopreventive drugs to overcome the side effects and resistance that arise from using chemical-based agents has garnered the attention of the scientific community. Polyphenol research is considered a promising field in the treatment and prevention of breast cancer.

## 1. Introduction

Currently, cancer is one of the most common life-threatening diseases worldwide, and breast cancer has the highest rate of diagnosis amongst women. There are three main strategies to block and postpone the stages of carcinogenesis [1,2,3]. The primary strategy considered is a preventive approach, which blocks the toxic, as well as the mutagenic, effects, which consequently inhibits tumor initiation and promotion. The secondary strategy presents anti-cancer potential during the early stages of carcinogenesis via various mechanisms, such as control of signal transduction, blocking angiogenesis, antioxidant mechanisms, hormones, and modulation of immunity, which finally result in the blockage of cancer progression. The third strategy for cancer treatment and prevention involves blocking the invasiveness and metastatic properties of a tumor via regulation of cell-adhesion molecules, protection of the extracellular matrix (ECM) from degradation, and up-regulation of genes that block metastasis [1,2].

The link between a diet that is rich in fruits and vegetables, and the prevention, as well as the reduction, of the occurrence of health-daunting diseases has been evidenced, and is partially ascribed to polyphenols [4,5,6]. The term polyphenol was first given to natural compounds bearing multiple (poly) phenol rings, which are widespread in various fruits, vegetables, wine, nuts, tea, coffee, and in many foods that are consumed daily by humans [7]. Polyphenols possess a broad spectrum of structural variations, which lead to a wide range of biological functions; among them, anti-cancer functions. Polyphenols possess a broad spectrum of structural variations in the carbon backbone chains, as well as alterations to primary and secondary structures due to methylation, glycosylation, and hydroxylation [6,8]. These structural variations may be responsible for their various health benefits, including antioxidant [9,10], anti-inflammatory, anti-angiogenic [11,12], and anti-proliferative mechanisms, as well as regulation of key signaling protein and enzyme functions [13].

## 2. Carcinogenesis: Overview and Molecular Basis

**Figure 1:**
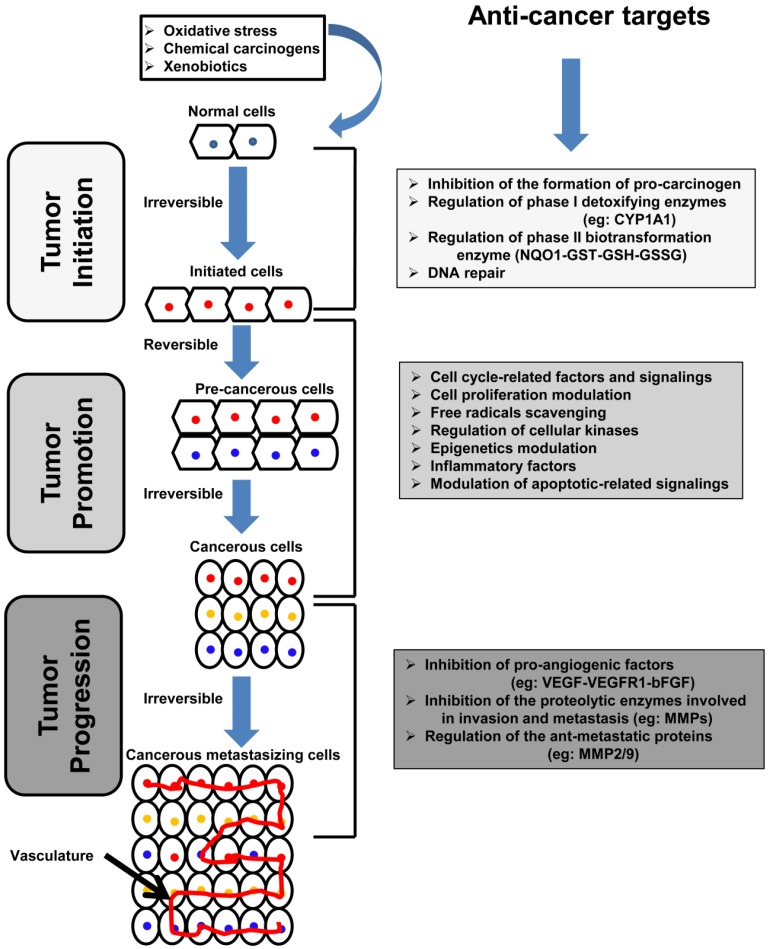
Schematic representation depicting the multistage process, including initiation, promotion, and progression, of carcinogenesis, and the biological targets of polyphenols at each step.

The tumorigenic process is complicated and occurs through a multistep procedure, including initiation, promotion, and progression, as illustrated in Figure 1 [14,15]. Initiation includes the entrance and distribution of cancer-causing agents in the cell, in particular, the nucleus, and interaction with DNA that finally results in the mutagenesis and emergence of the toxic effect [16]. This stage is irreversible, but can be prevented by phase I and phase II metabolizing enzymes, which transform the carcinogens into less toxic and soluble products [17,18].

Polyphenols present preventive effects against tumor initiation via numerous mechanisms, such as prevention of the formation of genotoxic molecules and blocking the activity of the mutagens-transforming enzymes [19,20]; regulation of heme-containing phase I enzymes, such as cytochrome P450s (CYPs) [21,22]; regulation of carcinogen-detoxifying phase II enzymes, such as NADPH-quinone oxidoreductase-1 (NQO1), quinone reductase (QR), glutathione *S*-transferase (GST), and uridine diphospho (UDP) glucuronosyl transferase (UGT) [23,24]; and prevention of the formation of DNA adducts [25].

The promotion stage, which takes time, is related to the proliferation of tumor-initiating cells. It is considered a reversible stage of tumorigenesis, and gives rise to pre-cancerous cells. The main features of this stage are cell proliferation and apoptosis. The tumor progression stage is the stage in which cells gradually transform to the malignant state. Metastasis and invasiveness also emerge during this stage, via the angiogenesis process, with the growth of new blood capillaries in the tumor, which is enhanced by the secretion of specific growth factors and growth factor receptors, such as platelet-derived growth factor (PDGF), PDGF receptor (PDGFR), vascular endothelial growth factor (VEGF), and VEGF receptor (VEGFR), leading to overgrowth and spread of the tumor [26].

Cathepsins, which belong to the lysosomal proteases superfamily, are implicated in tumor progression [27]. Cathepsin D, an aspartic protease, is considered to be a candidate as a clinical marker for breast cancer, and is involved in the activation of the inactive form of cathepsin B (procathepsin B) [28]. Cathepsin B is essential for the growth of breast cancer [29] and its down-regulation leads to a reduction of tumor progression [30]. The up-regulation of cathepsin B is an indicator of cancer progression and is a poor prognosis [31].

The urokinase plasminogen activator (uPA) system consists of serine protease uPA and various serine protease inhibitors, such as plasminogen activator inhibitors 1 and 2 (PAI-1 and PAI-2). Upon binding of uPA to urokinase plasminogen activator anchored receptor (uPAR), the activation of plasminogen takes place and leads to the production of the broad spectrum protease, plasmin [32]. Plasmin directly degrades the ECM, or indirectly via the activation of the zymogens of metalloproteinases (MMPs) [33].

There are numerous polyphenols that show potent inhibitory effects on the invasiveness and metastatic properties of cancer, which will be explained in detail in the following sections.

## 3. Overview on Breast Cancer and Cancer Stem Cells (CSCs)

Breast cancer represents about 25.2% of cancer cases in women, and commonly occurs in US women at a rate of one in eight cases [34,35]. In 2012, approximately 522,000 deaths were due to breast cancer [36]. Despite the success of emergent breast cancer therapeutics in decreasing mortality cases, the prognosis, in particular for the stage IV cancer, remains poor and needs further improvement [37]. The presence of small populations of cells with unique tumor recurrence and metastases represents a serious challenge during cancer therapy, and may be ascribed to the presence of a small population of specialized malignant cells, which are believed to be cancer stem cells (CSCs) [38,39].

In 2003, Al-Hajj et al. discovered the presence of CSCs in breast cancer [40]. They carried out fluorescence-activated cell sorting (FACS) analyses of primary breast cancer cells for the expression of the following markers, cluster of differentiation 44 (CD44), cluster of differentiation 24 (CD24), and epithelial specific antigen (ESA). They confirmed that CD44^+^ CD24^−/low^ cells possess the same characteristic features of CSCs, including self-renewal, differentiation, and high tumor induction properties [40].

## 4. Therapeutic Approaches to Breast Cancer and Development of Resistance

The main approaches for the treatment of breast cancer are surgical intervention, hormonal therapy, immunotherapy, chemotherapy, and radiotherapy. However, the recovery rate after application of these conventional methods is about 60%–80% for primary cancers and about 50% for metastatic ones [41,42].

Previously, the heterogenic features of cancer were calibrated, based on the following parameters: histological analysis, tumor grading, condition of lymph nodes, and specific markers, such as estrogen receptor (ER), progesterone receptor (PR), and, recently, human epidermal growth factor receptor 2 (HER2) [43]. Furthermore, tumor heterogeneity was verified using gene expression analysis and cDNA microarrays analysis [44].

There are four fundamental groups of patients with metastatic breast cancer that are subjected to treatment, including hormone receptor (HR)-positive patients, who are classified into two classes: luminal A type, with the highest invasiveness and the best prognosis, which is characterized by ER^+^, PR^+^, HER2^−^, and low ki67, and luminal B, which is characterized by ER^+^, PR^+^, HER2^+^ or HER2^−^, and high ki67 [45,46]. HR^+^ breast cancers have the best prognosis and can be treated with tamoxifen, an ER antagonist, fulvestrant, which directly hampers ER synthesis, and aromatase inhibitors, namely, anastrozole, exemestane, and letrozole [47].

HER2^+^, another subtype of metastatic breast cancer, is an ER^−^ breast cancer, and, therefore, is considered to be from the worst aggressive type of breast cancer [48,49]. To eliminate this type of cancer, various therapeutic strategies have been developed, such as a drug targeting HER2 receptor using humanized monoclonal antibodies, including trastuzumab (herceptin), pertuzumab, and lapatinib [49].

Triple-negative breast cancer (TNBC), voided of ER, PR, and HER2, is considered the worst type of metastatic breast cancer and has highly invasive properties, a large tumor size, poor prognosis, a high chance to relapse, is not responsive to hormonal therapy, and has lymph node involvement. There are several approaches to counteract TNBC, such as neoadjuvant chemotherapy, anthracyclines, taxanes, poly (ADP-ribose) polymerase protein (PARP) inhibitors, epidermal growth factor receptor (EGFR) inhibitors, and platinum-containing chemotherapeutic agents [50,51,52].

Chemotherapy remains a crucial approach for cancer management in all patient groups. However, HER2-positive tumor patients, and patients with TNBC, need endocrine therapy in addition to chemotherapy [53]. Taken together, new, alternative therapeutics, in particular, natural products, need to be explored using mammosphere culture in order to overcome this problem.

## 5. Overview on Polyphenols

**Figure 2:**
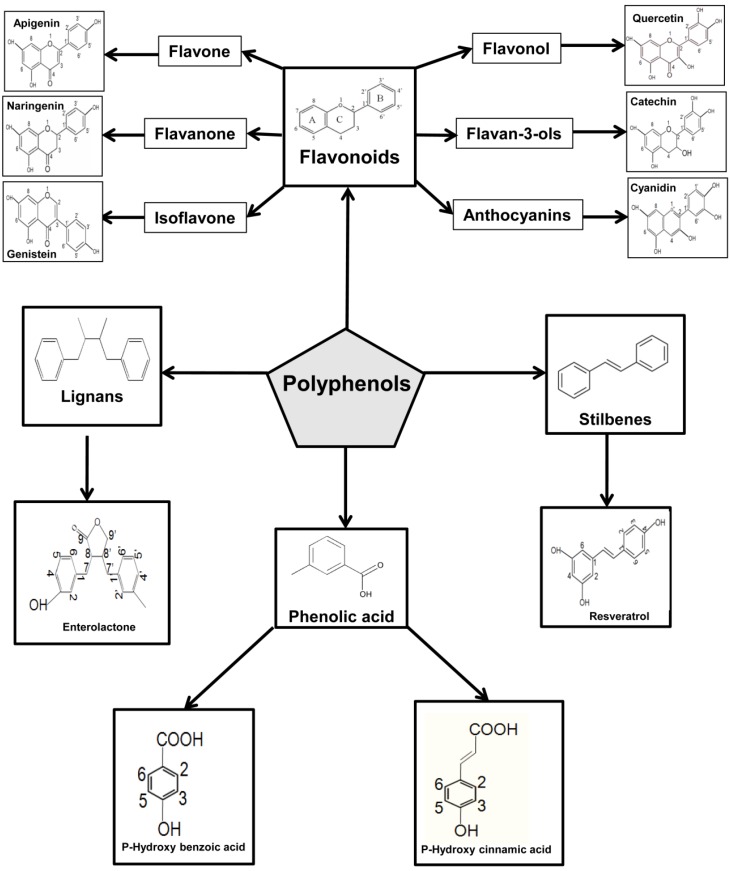
Diagram summarizing the classes of polyphenols and their basic chemical structures. Polyphenols can be separated into two main classes: flavonoids and non-flavonoids. The flavonoid class consists of two benzene rings, linked by a heterocyclic pyrone C-ring. The non-flavonoids class contains more intricate molecules, namely, benzoic acid, hydroxycinnamates, stilbenes, lignans, gallic acids tannins, and gallotannins.

Polyphenols, a broad category of natural compounds and plant metabolites, possess one, or numerous, benzene rings that bear one, or several, hydroxyl groups. They are considered to be complicated antioxidants that are abundantly present in our daily diet, in particular, they can be found in fruits, legumes, spices, cocoa, vegetable, coffee, nuts, beer, wine, and olive oil [54]. Average daily consumption of polyphenols is estimated to be around one gram [55]. In nature, polyphenols generally exist conjugated with organic acids and sugars, and, accordingly, can be classified into two main categories; flavonoids and non-flavonoids, as shown in Figure 2.

The flavonoid category consists of two benzene rings, linked by a heterocyclic pyrone C-ring, and the non-flavonoid category contains more complicated molecules (benzoic acid, hydroxycinnamates, stilbenes, lignans, gallic acids tannins, and gallotannins) [56,57].

Polyphenols have been reported to possess special activities that are beneficial for human health, such as anti-oxidant [58], anti-infection [59,60,61], anti-cancer [62,63], neuroprotective [64], and anti-inflammatory [65] effects. Their broad activity could be attributed to several mechanisms, including interaction with, as well as modulation of, a wide range of proteins, enzymes, and membrane receptors, regulation of gene expression, apoptosis induction, vasodilatation, and modulation of cell signaling pathways [66,67,68,69,70].

There are similarities between some groups of flavonoids, such as isoflavones and lignans, and the estrogens, and, accordingly, they are considered as a phytoestrogen. Their anti-estrogenic activity has been exploited and applied in a wide range of studies [71]. Especially, the potent anti-cancer activity of polyphenols can be ascribed to their targeting of aromatase, antioxidant mechanisms, anti-inflammatory mechanisms, and anti-estrogenic mechanisms [72,73,74,75,76].

Flavonoids are considered to be the largest category of the polyphenols, and are characterized by their low molecular weight [77,78]. The structural characteristics of flavonoids can determine their functions and bioavailability, and can be used for classification into various groups.

The basic structure of flavonoids consists of a flavan nucleus (2-phenylchroman) containing 15 atoms that constitute three rings (A-ring (C6), B-ring (C6), and C-ring (C3)). The variation among flavonoids depends on the following: changes in the C-ring (presence of the 3-hydroxyl group, and double bond or 4-oxo group) and changes in the A- and B-rings, such as the difference in the number and the position of the hydroxyl and methoxyl groups. If one or more sugar group binds to the flavonoid structure, they are called “flavonoid glycosides”, whereas flavonoids without a sugar group are described as “aglycones”.

Dietary flavonoids are mainly “flavonoids glycosides”, except for flavanols. Moreover, our research group revealed various aspects of the biological activities and health benefits of numerous flavonoids, such as antioxidant, antiviral, and anti-cancer properties, which were evidenced in vitro and in vivo [79,80,81,82,83,84].

**Figure 3:**
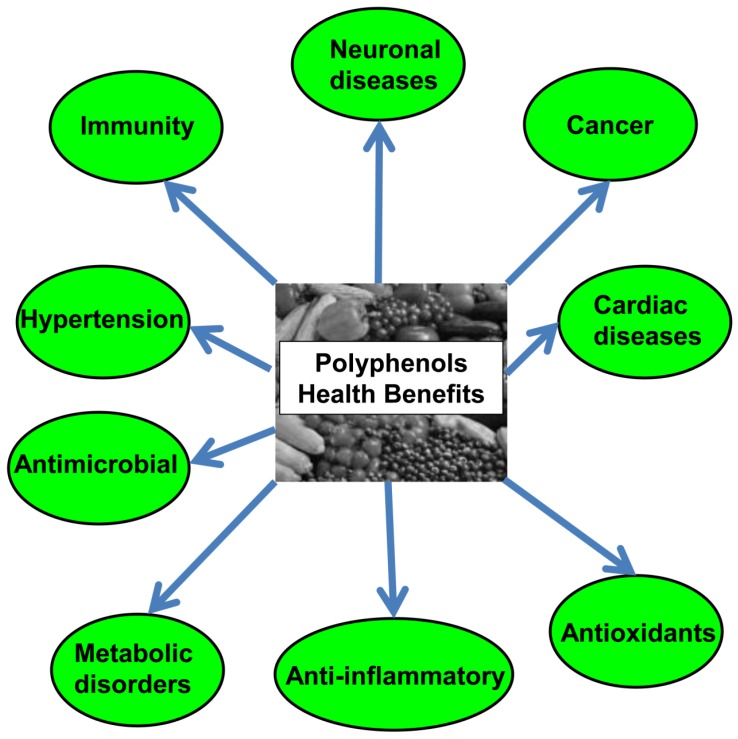
Overview summarizing the main health benefits of polyphenols. Polyphenols play key roles in the prevention of serious diseases that threaten human life and negatively affect quality of life, such as cancer, metabolic diseases, neurodegenerative diseases, hypertension, and cardiac diseases.

Polyphenols are considered the main natural antioxidant component in fruits, vegetables, tea, oils, and cereals. The wide range of health benefits of dietary polyphenols is ascribed to their potential in reducing the risk, as well as preventing, serious diseases, such as cancer, metabolic diseases, neurodegenerative diseases, and heart diseases, which threaten human life and negatively affect quality of life, as summarized in Figure 3 [85].

There is a large body of literature that describes the impact of polyphenols on human health and disease prevention [86,87]. Polyphenols are present in foods as intricate combinations of various chemical formulations of several polyphenol compounds, such as oligomers, chlorogenic acid, hydroxycinnamic acids, and epicatechin (in apples) [88]. Moreover, these dietary polyphenols are present in combination with sugar residues that conjugate with hydroxyl groups and aromatic carbons, can be combined with organic and carboxylic acids, and with amines [89]. In cereals, polyphenols are conjugated with polysaccharides of the cell wall [90], and in fruits, the amount of conjugated polyphenols is much higher than the amount of free polyphenols [91].

The absorption rate and site of polyphenols are modulated by their structures [92]. For instance, glycosides can be absorbed in the small intestine, except for glycosides that link to the rhamnose group metabolized by the enzyme, α-rhamnosidase, which is secreted by microflora in the colon [93]. Glycosides can be metabolized by several enzymes, including cytosolic β-glucosidase and the membrane-located lactase phlorizin hydrolase [94,95].

On the other hand, the acylated polyphenol compounds, flavan-3-ols (epicatechin), are absorbed directly into the enterocyte without hydrolysis [96]. Hydroxycinnamic acids, which are esterified with organic acids, lipids, and sugar, are partially absorbed in the small intestine, and a major portion is metabolized by colonic microflora. The colon is considered a suitable site for the absorption of polymeric proanthocyanidins.

## 6. Correlation between Polyphenols’ Anti-Cancer Activity and Autophagy

Autophagy is a cellular phenomenon that occurs as a response reaction against stress factors, such as starvation, oxidative stress, and toxicity [97]. During the autophagy process, catabolic lysosomal degradation takes place in order to maintain cellular homeostasis.

Autophagy-related genes (ATG) and their proteins are essential for the formation of the double-membrane vesicles needed for the engulfment of damaged cellular organelles in the cytosol. Beclin-1 (Atg6 in yeast), which is located on human chromosome 17q21, is considered one of the key components of ATG proteins. It exhibits haploinsufficiency, and its identification may have unveiled a crosslink between autophagy and human cancer. Its monoallelic deletion has been detected in breast, ovary, and prostate cancers [98,99].

The crosslink between diet and autophagy is well-known, and dietary restriction or starvation are related to autophagy induction and influence on health [100,101]. Autophagy induction is modulated by the level of cellular ATP and energy, which are detected by the cellular energy sensor, adenosine monophosphate kinase (AMPK). AMPK activation is enhanced as a response to the low ratio of ATP/AMP and nutrient deprivation via its upstream kinase, liver kinase BQ (LKB1 kinase). AMPK inhibits the activity of the mammalian target of rapamycin 1 (mTORC1) directly via phosphorylation of RAPTOR, or indirectly through activation of TSC1/2, which enhance the activity of GTP-Rheb [102,103]. Inactivated mTOR is involved in autophagy induction via activation of complexes, including ULK1, Atg13, and the FAK-family interacting protein of 200 kDa (FIP200) [104].

**Figure 4:**
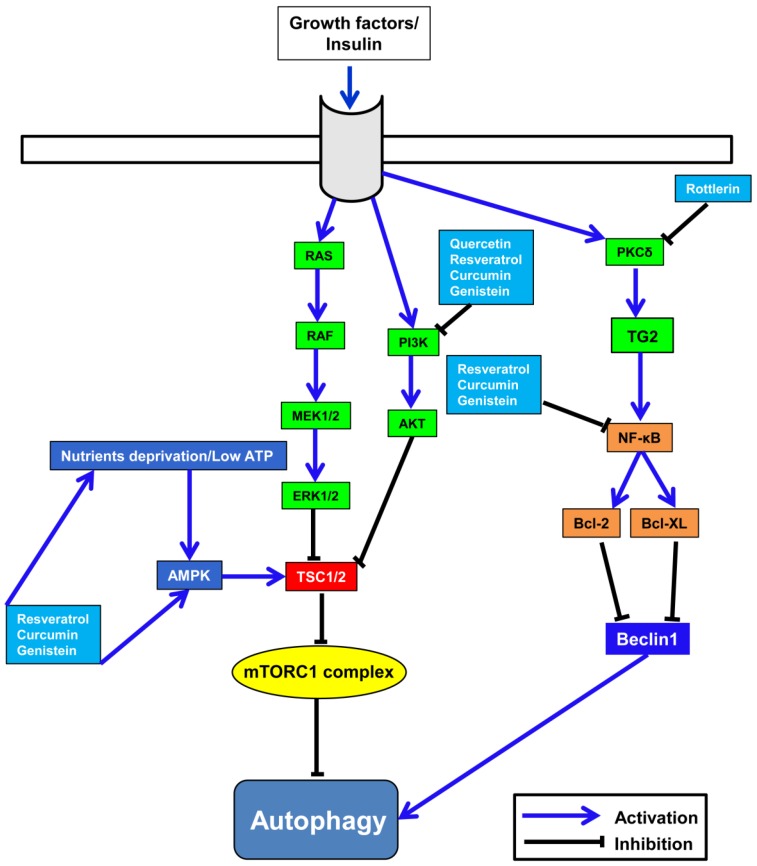
Role of polyphenols in the modulation of autophagy in breast cancer. Polyphenols modulate the autophagy process by regulating various signaling pathways, such as the PI3K/AKT, RAS/RAF/ERK, PKCδ, and AMPK signaling pathways.

Below, we will discuss examples of polyphenols, and how autophagy signaling pathways and transcription factors are involved in their anti-cancer potentials, as summarized in Figure 4.

### 6.1. Resveratrol

Resveratrol (3,4′,5-trihydroxy-trans-stilbene), the main polyphenol in grapes and peanuts, exists in red wine at a concentration of about 0.1–1.8 g per 100 mL. In mice, resveratrol potently mitigates the harmful consequences of a high-fat diet that influences longevity and lifespan [105]. This lifespan-increasing effect is attributed to the activation of sirtuin (SIRT1) via an autophagy-mediated mechanism [106]. The crosslink between SIRT1 and autophagy is attributed to the potency of SIRT1 to deacetylate the core elements, such as Atg5, Atg7, and Atg8, of autophagy induction [107]. Resveratrol is a well-known polyphenol modulating SIRT1 [108]. The anti-cancer activity of resveratrol has been proven in vitro and in vivo [109,110,111], and is mediated by numerous mechanisms, such as apoptosis, cell cycle arrest, kinase signaling pathways, and autophagy [109,112].

The implication of resveratrol in the induction of autophagy via the accumulation of autophagosomes has been proved in various cell lines [109,113,114]; however, resveratrol treatment induces non-canonical autophagy, which is independent of Beclin-1, vacuolar protein sorting 34 (Vps34), and Atg-dependent autophagy in breast cancer cells [115].

Apoptosis-resistant cell lines, such as breast cancer MCF-7 cells, which are deficient in caspase-3, showed sensitivity to resveratrol treatment, and, interestingly, activation of caspase-9, as well as chromatin condensation, were detected in resveratrol-treated MCF-7 cells [116].

Recently, FoxO transcription factors have been shown to play an important role in apoptosis and autophagy induced by resveratrol treatment [117]. In human colorectal cancer, resveratrol-induced cell death was abolished upon genetic inhibition of the function of autophagy-related proteins, including PI3K, Lamp2b, and Beclin1 [113]. In human epidermoid carcinoma cells, exposure to resveratrol led to a decrease in the expression level of Rictor protein, and of mTORC2, and ultimately a reduction of RhoA-GTPase [118].

Reactive oxygen species (ROS) mediate the significant up-regulation of AMPK upon resveratrol treatment in etoposide-resistant HT-29 colon cancer cells, and, in turn, augment the potential of etoposide to induce apoptosis [119]. In addition, resveratrol exposure increased ROS generation and cleavage of caspase-8 and caspase-9, and ultimately induced autophagy via up-regulation of microtubule-associated protein 1 light chain 3-II (LC3-II) expression in colon cancer [120].

Resveratrol leads to autophagy induction via the up-regulation of p62/sequestome-1 (SQSTM1), and AMPK/mTOR-mediated, by JNK in imatinib-sensitive and imatinib-resistant chronic myelogenous leukemia cells (CML) K562 [121].

### 6.2. Silibinin

Silibinin, which is a flavonolignan extracted from milk thistle (*Silybum marianum*), possesses protective effects for the liver [122] and neurons [123,124]. Recently, the anti-cancer activity of silibinin has been demonstrated in vitro and in vivo [125,126,127]. In human colon cancer cells, silibinin treatment led to activation of the extrinsic (receptor-related) and intrinsic (mitochondria-related) apoptosis pathways, as well as activation of the autophagic process [128]. Pharmacological inhibition of autophagy with treatment of bafilomycin-A1 (Baf-A1) in silibinin-exposed human colon cancer cells resulted in autophagy inhibition, which is accompanied by activation of cell death. Accordingly, silibinin treatment of human cancer cells induced cytoprotective autophagy, and ROS was a mediator in silibinin-induced apoptosis and autophagy in tumor cells [129,130]. On the other hand, the ROS-scavenging activity of silibinin was also shown in vitro and in vivo [123,131].

An interesting study demonstrated the potential of silibinin to induce autophagic cell death in breast cancer cells. This effect was confirmed by high expression of LC3-II, increase of Beclin-1, high Atg-12-Atg-5, and down-regulation of Bcl-2 [132]. Upon treatment with pharmacological inhibitors of autophagy, 3-methyladenine (3-MA) and Baf-A1, silibinin-induced breast cancer cell death was mitigated. Silibinin treatment led to ROS generation, which was correlated with the disruption of mitochondrial membrane potential and ATP depletion, which were further blocked by treatment of *N*-acetyl cysteine (NAC) and ascorbic acid [132].

Of note, silibinin-exposed breast cancer cells showed up-regulation of Bcl-2 adenovirus E1B 19-kDa-interacting protein 3 (BNIP3). Small interfering RNA (siRNA) targeting BNIP3 abrogated silibinin-induced cell death, ROS generation, ATP depletion, and the disruption of mitochondrial membrane potential [132].

Silibinin-induced autophagy and apoptosis in MCF-7 cells are concomitant with the down-regulation of AKT, mTOR, and ERK [133]. Co-treatment of ERα antagonist, methyl-piperidinopyrazole (MPP) dihydrochloride, with silibinin led to the aggravation of the apoptosis and autophagy induced by silibinin treatment. These results indicate that ERα inhibition by silibinin mediates the down-regulation of AKT, mTOR, and ERK, and the final induction of apoptosis and autophagy in MCF-7 cells [133].

### 6.3. Quercetin

Quercetin (3,3′,4′,5,7-pentahydroxyflavanone), a flavonol, exists in a wide range of fruits and vegetables, such as onions, apples, and berries, and is considered one of the most common antioxidants in the human diet [134]. The application of quercetin to inhibit tyrosine kinase has been approved for clinical trials [135]. The anti-cancer potential of quercetin has been shown in various in vitro and in vivo studies [136,137,138,139]. Down-regulation of mTOR activity, and the subsequent formation of autophagosomes by quercetin treatment, have been evidenced [140].

In gastric cancer cells, quercetin induced cytoprotective autophagy that was abrogated upon treatment with the lysosomal inhibitor, chloroquine, or silencing of Atg5 or Beclin-1 using siRNA, and led to apoptotic cell death [141].

Hypoxia-induced factor 1α (HIF-1α) and Akt-mTOR signaling pathways are mediators of quercetin-induced cytoprotective autophagy. The components of the mTOR signaling pathway, in particular, mTORC1, play key roles in the maintenance of cellular homeostasis via modulation of protein synthesis through p70S6 kinase, which activates the ribosomal S6 subunit, and phosphorylation of 4E-BP1 (eIF4E binding protein 1) that inhibits the sequestration of the eukaryotic initiation factor of protein biosynthesis (eIF4). In various cancer cell lines, quercetin modulates the mTOR signaling pathway through down-regulation of the phosphorylation level of the ribosomal S6 subunit via p70S6 kinase, as well as via activation of 4E-BP1 [140].

### 6.4. Genistein

Genistein (4′,5,7-trihydroxyisoflavone), an isoflavone, is widely distributed in soybean and presents a broad spectrum of in vitro and in vivo anti-cancer potential in numerous cancer cells, through cell cycle arrest, induction of apoptosis, blocking of angiogenesis, inhibition of telomerase activity, and blocking inhibition of DNA topoisomerase II [142,143,144,145].

In ovarian cancer cells, genistein treatment led to cell death, which is independent of caspase signaling pathways and induced autophagy [146]. The autophagy induced by genistein treatment can be recovered upon treatment with methyl pyruvate, the substrate for oxidative phosphorylation and fatty acid synthesis.

Genistein-exposed ovarian cancer cells showed a marked reduction in glucose uptake that may be attributed to the inactivation of AKT signaling [146]. Inhibition of the aggregate that is formed by the interaction between cyclic AMP phosphodiesterase-4A4 (PDE4A4) and SQSTM1 protein (p62) is essential for the induction of autophagy. This can be explained by the role of SQSTM1 protein in interacting with LC3, which has a pivotal role in vesicle formation in autophagosomes [147]. Genistein-treated ovarian cancer cells showed marked autophagy due to inhibition of the formation of PDE4A4 and SQSTM1 aggregates, activated by ERK and PKC inhibitors [148].

### 6.5. Curcumin

Curcumin, diferuloylmethane extracted from *Curcuma longa*, is the key constituent of turmeric, and possesses various biological functions with minimal toxicity, such as antioxidant, anti-inflammatory, and anti-cancer functions [149,150]. In malignant glioma cells, curcumin exposure led to cell cycle arrest and autophagy induction through up-regulation of the ERK1/2 signaling pathways and down-regulation of the Akt/mTOR/p70S6K signaling pathways [151]. In bladder cancer cells, curcumin dephosphorylated AKT, and, in turn, activated LC3-II [152].

The autophagy-inducing capacity of curcumin was exploited in cellular protection against oxidative stress-induced cell death in human umbilical vein endothelial cells. This was mediated by modulation of the autophagy machinery, including activation of LC3-II, inhibition of PI3K/Akt/mTOR core signaling, and promotion of FOXO1 (autophagy mediator) [153]. In curcumin-exposed human colon cancer cells, there was a significant increase in the conversion of LC3-I to LC3-II, as well as degradation of SQSTM1 [154]. These effects were markedly abrogated after treatment with an ROS scavenging compound, NAC, indicating that ROS is a mediator of curcumin-induced autophagosome formation and cell death [154].

In malignant glioma cells, curcumin treatment induced autophagy that is attributed to the up-regulation of ERK signaling, which is concomitant with the down-regulation of the Akt/mTOR/p70 ribosomal protein S6 kinase (p70S6K) pathway [155]. Moreover, SIRT1 was modulated by curcumin in the regulation of autophagy and other cellular events [108].

Curcumin remarkably enhanced the expression of AMPK, accompanied by p38 signaling-mediated cell death in ovarian cancer cells [156]. Similarly, curcumin induced ROS generation at the beginning of apoptosis and autophagy in oral squamous cell carcinoma, and NAC treatment abolished curcumin-mediated autophagosome formation [157]. In addition to the induction of autophagy, curcumin exposure led to apoptosis via inactivation of Bcl-2 protein and down-regulation of NF-κB in cancer cells [158,159].

### 6.6. Rottlerin

Rottlerin, also called mallotoxin, is one of the active components of the Kamala tree (*Mallotus philippensis*), which grows widely in Southeast Asia. In 1994, the pharmacological effects of rottlerin were revealed, after its potential to specifically inhibit the activity of protein kinase C delta (PKCδ) was demonstrated [160]. Therefore, the potency of rottlerin to block PKCδ activity has been exploited in various biological functions related to PKCδ [161].

Recently, rottlerin was shown to exhibit various biological activities, including human T-cell response inhibition [162], potassium channel activation [163], in vitro and in vivo neuroprotection [164], antioxidant activity [165], antihistaminic activity [166], and anti-cancer activity [167]. The crosslink between tissue transglutaminase (TG2) and NF-κB was evidenced [168]. Moreover, implication of NF-κB in the autophagy process was proven [169].

In pancreatic cancer cells, rottlerin, as well as PKCδ siRNA treatment, led to a drastic decrease in cell proliferation, which was accompanied by a significant reduction in mRNA and protein levels of TG2, without showing any apoptotic changes [170]. However, rottlerin-treated pancreatic cancer cells showed significant autophagy, which was evidenced by cytoplasmic acidic vacuoles and the up-regulation of LC3-II, similar to that of TG2-specific siRNA-treated cells. Belin-1 knockdown abrogated the potential of rottlerin and TG2 siRNA to induce autophagy in pancreatic cancer cells.

In human pancreatic CSCs, rottlerin treatment led to early autophagy, evidenced by the formation of autophagosomes, LC3-II formation, up-regulation of Atg7 and Beclin-1, as well as down-regulation of the pro-apoptotic proteins, Bcl-2 and Bcl-X_L_ [171]. Treatment of 3-MA or genetic inhibition of autophagy via silencing of the autophagy-specific genes, Atg7 and Beclin-1, blocked the potential of rottlerin to induce autophagy and enhanced rottlerin-induced apoptosis [171].

In human breast cancer cells, rottlerin treatment showed TSC2-dependent inhibition of the mTORC1 signaling pathway and the accumulation of autophagosomes as a consequence [172]. Taken together, we described the mechanisms of polyphenols in autophagy modulation in terms of their anti-cancer functions. However, these findings need to be scrutinized in depth with respect to breast cancer and in vivo using animal models that possess genetic modifications of autophagy-related genes.

## 7. Anti-Cancer Activity of Polyphenols against Breast Cancer: Molecular Mechanisms

**Figure 5:**
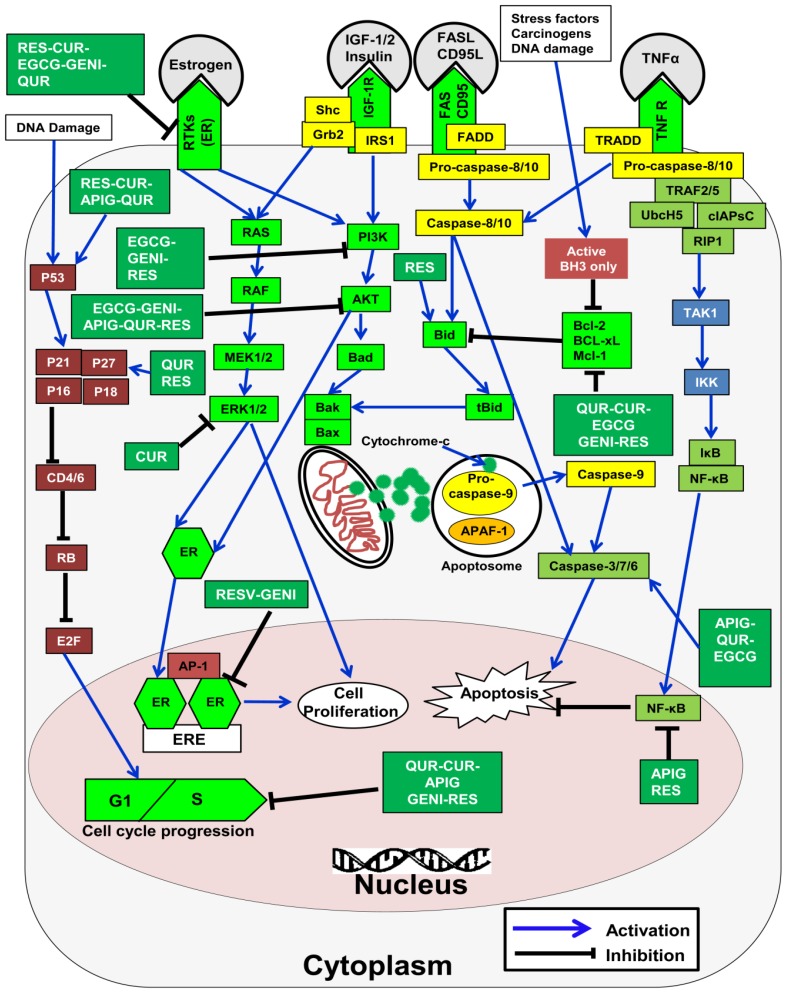
Comprehensive representation summarizing the possible mechanisms of action of polyphenols against breast cancer. The anti-cancer activity of polyphenols is mediated via the regulation of various signaling pathways, such as intrinsic and extrinsic apoptotic pathways, estrogen-related signaling pathways, cell cycle arrest, and inflammation-related signaling pathways. RES, resveratrol; CUR, curcumin; GENI, genistein; QUR, quercetin; APIG, apigenin.

The anti-cancer activities of polyphenols against a wide range of cancers, such as breast cancer [173], prostate cancer [174], colorectal cancer [175], pancreatic cancer, lung cancer, colorectal fibrosarcoma, and leukemia, have been proven [176]. The possible mechanisms underlying the anticancer activity of polyphenols against breast cancer are summarized in Figure 5, and the possible molecular mechanisms by which polyphenols kill breast cancer are described below.

### 7.1. Modulation of ROS

In fruits, polyphenols represent a major portion of the antioxidants compared to vitamin C [177]. Antioxidant activity is one of the key mechanisms that contribute to the protective effect of polyphenols against oxidative damage.

Cellular redox balance can be maintained by cellular antioxidant enzymes, including superoxide dismutase (SOD), peroxiredoxins (PRXs), catalase (CAT), glutathione peroxidase (GPx), and glutathione reductase (GR) [178]. However, mitigation of excessive generation of ROS by the cellular antioxidant enzymes is difficult [179].

Polyphenols are directly involved in the reduction of the Fenton reaction, via chelation of iron, thereby protecting cells from oxidation from highly reactive hydroxyl radicals [180,181,182]. The potent antioxidant activity of polyphenols is attributed to their ability to scavenge a broad spectrum of highly reactive species, such as ROS, reactive nitrogen species (NOS), chlorine species, peroxynitrous acid (ONOOH), and hypochlorous acid (HOCl) [183]; they also block the chain reactions of lipid peroxidation (chain breakers) as a consequence [180,184]. The antioxidant activity of flavonoid compounds is also mediated by targeting NFκB- and MAPK-related signaling pathways [185]. Polyphenols can work as co-antioxidants, as they show synergistic activity with other antioxidants, such as α-tocopherol (vitamin E), leading to the regeneration of vitamin E [186].

The polyphenols structures, such as the hydroxyl group’s number and position, hydroxylation degree, and distance between the aromatic ring and the carbonyl group, play a pivotal role in its antioxidant activity and metal chelating property. For instance, within the flavonol group, quercetin showed the most potent antioxidant activity due to its 3-hydroxy group [187]. Additionally, polyphenol potential for metal chelation and scavenging of free radicals could be elevated with a B-ring bearing catechol moiety, C-ring bearing 4-oxo group, and the presence of a double bond [188].

Cinnamic acid and its derivatives showed relatively better antioxidant properties compared to benzoic acid, due to the longer distance between the aromatic ring and the carbonyl group. Additionally, the presence of the hydroxyl group at the para and/or ortho position on the benzoic ring enhances antioxidant potential compared to the presence of the hydroxyl group at other positions [189].

Biochanin A, an isoflavonoid purified from red clover (*Trifolium pratense*) showed preventive activity against the incidence of mammary gland cancer, after exposure to carcinogenic agents in prepubertal rat [190]. It potentially counteracted oxidative stress through a significant up-regulation of SOD, CAT, GPx, GST, and DT-diaphorase (DTD), as well as a remarkable reduction of lactate dehydrogenase (LDH) and lipid peroxidation (LPO) activities.

The protective action of resveratrol against 17β-estradiol (E2)-induced carcinogenesis was evidenced in vitro and in vivo, and was mediated by a significant increase in the expression of nuclear factor erythroid-related factor-2 (Nrf-2), which consequently up-regulated the expression of antioxidant genes, including NQO1, SOD3, and 8-oxoguanine DNA glycosylase 1 (OGG1) [191].

Green tea is composed of four main catechins, (−)-epicatechin (EC), (−)-epicatechin gallate (ECG), (−)-epigallocatechin (EGC), and (−)-epigallocatechin-3-gallate (EGCG). EGCG, the most abundant polyphenolic catechin, is considered the most active catechin, possessing various biological functions in vitro and in vivo [15,192]. Low concentrations of EGCG resulted in significant reduction in ROS generation, which was induced on exposure to environmental carcinogens [193]. However, it had no significant effect on the regulation of the antioxidant enzymes (SOD and CAT) in MCF-7 breast cancer cell lines, but showed up-regulated expression of NQO1, the main detoxification enzyme of phase II [193].

Genistein showed significant antioxidant action and better mitochondrial function in T47D with low ERα/ERβ ratios, whereas no significant antioxidant effect was shown in MCF-7 cells with high ERα/ERβ ratios [194]. Therefore, ERβ is essential for the antioxidant potential of genistein [194].

Curcumin-treated breast cancer cells showed a significant decrease in cell proliferation, mediated by Nrf-2 nuclear translocation, associated with the down-regulation of Flap endonuclease 1 (Fen1), which is a nuclease involved in DNA repair [195]. It also showed ROS scavenging actions in MCF-7 breast cancer cells exposed to nickel oxide nanoparticles [196].

On the other hand, polyphenols presented a pro-oxidant action that was determined by the application of high concentrations, or the presence of, metal ions that mediate the formation of chelates and the oxidation of polyphenols [197,198,199]. The pro-oxidant effects of polyphenols are involved in their anti-cancer activity. For example, the pro-oxidant activity of polyphenols was correlated with mitochondrial dysfunction and DNA damage mediated by high oxidative stress, and, in turn, resulted in apoptosis [200,201]. In breast cancer cells, 50 µM of soy isoflavone, genistein, showed a pro-oxidant action via mobilization of copper ions that led to DNA damage, an increase in ROS generation, and apoptosis [202]. The pro-oxidant effect of curcumin via ROS generation, in a time-dependent manner, in MCF-7 and MDA-MB-231 breast cancer cell lines, was demonstrated [203]. Additionally, high concentration of EGCG showed a marked increase in ROS generation in Hs578T breast cancer cells [204]. In vivo studies are needed to confirm, as well as explain, the contradictory findings of the antioxidant and the pro-oxidant effects of polyphenols.

### 7.2. Modulation of Inflammation-Related Factors

Cancer occurs at sites of chronic inflammation, and is proved by the presence of inflammatory cells in cancer [205]. For instance, inflammatory responses from microbial infection represent 15%–20% of cancer death cases worldwide [205], and, therefore, non-steroidal, anti-inflammatory drugs are one option to mitigate cancer deaths arising from inflammatory responses [206,207]. Chronic inflammation can give rise to an aggressive type of breast cancer, inflammatory breast cancer (IBC), which represents 5% of breast cancers and is associated with 8%–10% of breast cancer deaths [208,209].

Polyphenols from blueberry powder present potent in vitro and in vivo inhibitory properties against breast cancer proliferation and metastasis by regulation of interlukin-6 (IL-6) [210]. Polyphenol-enriched blueberry preparation (PEBP) potently inhibited breast cancer proliferation, cell movement, and migration, by targeting inflammatory signaling cascades, including the ERK, AKT, and STAT3 pathways [211]. In this regard, the anti-inflammatory activity of polyphenols may be important mechanisms underlying their anti-cancer and chemopreventive potentials. The anti-inflammatory activity of polyphenols is attributed to their ability to block properties against NF-κB [212], cyclooxygenase (COX-2) [213], and lipoxygenase (LOX) [214] activities.

NF-κB plays a pivotal role in the control of the expression level of inflammation-related cytokines, TNFα and IL-1 [215], as well as up-regulation of COX-2, which is an inducible prostaglandin G/H synthase that is highly expressed in numerous tumor cells [216]. The possible mechanisms by which dietary polyphenols block the up-regulation of NF-κB involve the inhibition of phosphorylation and/or proteasomal degradation of IκBs, inhibition of the liberation of NF-κB dimers from the cytoplasm into the nucleus, and hampering the interaction between NF-κB and target DNA [217,218]. Curcumin [219], green tea rich polyphenols [220], quercetin [221], and resveratrol [222] showed potent anti-cancer activities by blocking the expression level of NF-κB.

The potential of curcumin to inhibit cancer metastasis has been confirmed in vitro using breast cancer cells, as well as in vivo, using immunodeficient mice. In this study, the authors showed the crosslink between curcumin and the inhibition of the expression level of MMPs via down-regulation of the expression level of NF-κB and transcription factor AP-1, as well as inhibition of the phosphorylation of NF-κB, in turn, reducing the phosphorylation of ΙκB and p65 [223]. The anti-metastatic action of curcumin in breast cancer cells is explained by its inhibition of the nuclear translocation of NF-κB via dephosphorylation of IκB, resulting in the down-regulation of inflammation-related cytokines, such as CXCL1/2 [224].

Green tea catechin, EGCG, stimulated apoptosis in γ-radiation-exposed breast cancer cells, and was associated with the inactivation of NF-κB [225]. Combined treatment with EGCG and curcumin potently reduced the expression of the BCSC marker, CD44, via dephosphorylation of STAT3, and, in turn, prevented its nuclear translocation and its interaction with NF-κB for activation of target transcription factors [226].

The activation of STAT3 is essential for the proliferation and metastasis of a wide range of cancer, and its high expression is indicative of a poor prognosis. Targeting the STAT3 pathway is considered one of the key therapeutic approaches to block cancer proliferation and metastasis [227,228]. The inhibitory activity of silibinin against the phosphorylation of STAT3 has been demonstrated in preclinical studies in various cancers [229]; however, further clinical trials are needed to fully characterize silibinin activity as a STAT3 inhibitor. 

In nude mice inoculated with MCF-7 cells, oral administration of xanthohumol, a prenylated flavonoid that was purified from hops (*Humulus lupulus* L.), resulted in a significant reduction in infiltration of mononuclear and polymorphonuclear inflammatory cells, an increase in the percentage of apoptosis, a reduction in the density of microvessels, and a decrease in nuclear and cytoplasmic NF-κβ expression and cytoplasmic staining of Pi-Iκβα, compared to tumors in untreated control mice [230].

### 7.3. Modulation of the Estrogen Receptor

Estrogens are a commonly-listed human carcinogen, and high exposure to estrogen is highly related to the incidence of breast cancer, via increased cell proliferation through interaction with ER [231]. Patients with breast cancer show a high level of estrogen in the circulating blood [232]. Simply, breast cancer could be treated by inhibition of this action, as well as the production of estrogens, or interference, in the binding to ER [233,234]. ER targeting can be performed using classical drugs, such as raloxifene and tamoxifen, which are collectively called selective estrogen receptor modulators (SERMs) and are effectively applied in pre-and post-menopausal women [235].

Two types of ER, ERα and ERβ, are differentially expressed in organs, and ERα is highly expressed in the uterus and is involved in the proliferation of the endometrium, whereas ERβ is abundant in mammary glands, ovary, and the hypothalamus [236]. ERβ was involved in the induction of various transcription factors that are related to the modulation of cell proliferation and death, the cell cycle, and differentiation [237,238].

Owing to the similarity in the structure of non-steroidal compounds or phytoestrogens and E2, several phytoestrogens were shown to bind to ERα and ERβ. The binding affinity of genistein to ERβ is about 7–48-fold higher than to ERα [239,240,241]. In contrast, a flavonoid, xanthohumol, showed potent anti-cancer activity against luminal-type breast cancer by inhibiting the interaction between the growth of luminal-type guanine nucleotide-exchange protein 3 (BIG3) and tumor suppressor prohibitin 2 (PHB2) [242]. The released PHB2 binds to the nuclear and cytoplasmic ERα, and blocks E2-associated signaling pathways, thereby inhibiting the proliferation of ERα-positive breast cancer cells in vitro and in vivo.

The flavonoid compound, ellagic acid, which is widely distributed in berries, grapes, and nuts, possesses phenolic rings and ortho-dihydroxyl groups involved in the recognition of ER receptors [243]. Ellagic acid significantly reduced cancer size and occurrence in ACI rats exposed to estrogen with decreased CYP1A1 activity [244].

Similar to most flavones, including fisetin, apigenin, and kaempferol, morin (3,5,7,2′,4′-pentahydroxyflavone), a flavonol compound that is found in copious amounts in onion, mill (*Prunus dulcis*), and fig (*Chlorophora tinctoria*), showed strong inhibitory effects against oxidative stress [245]. Morin possesses hydroxyl groups in the 7- and 4′-positions, parallel to the 3- and 4′-positions, on synthetic estrogen diethylstilbestrol (DES), and is therefore considered a phytoestrogen [246].

Luteolin (3′,4′,5,7-tetrahydroxyflavone) potentially decreased the expression of insulin-like growth factor-1 (IGF-1), which correlated with MCF-7 proliferation. This effect was attributed to the capacity of luteolin to down-regulate ERα expression [247]. Knockdown of ERα led to the abolishment of the potency of luteolin to inhibit MCF-7 cell proliferation.

The chemical structure (2 hydroxyl groups and phenolic ring) of quercetin is akin to the structure of estrogen and it is considered a phytoestrogen that potentially binds to ER and modulates cell cycle progression. It also presents anti-cancer actions via estrogen-related pathways [248,249].

Resveratrol inhibits the growth of various breast cancer cells (MCF-7 and MBA-MB-231) via modulation of the expression level of various transcription factors associated with cell cycle regulation, apoptosis, metastasis, and angiogenesis. These actions were more pronounced in ER^+^ cells than in ER^−^ cells, assuring the importance of the binding to ER in the enhancement of the anti-cancer activity of resveratrol against breast cancer [250].

In ERα-positive MCF-7 cell lines, the physiological dose of EGCG induced a significant reduction in cell growth, which was correlated with the reduction in the protein levels of ERα and IGF-1 receptor (IGF-1R), as well as the up-regulation of p53 and p21 [251]. Whereas, in ERα-positive T47D cell lines expressing mutated p53, EGCG treatment had no significant inhibitory effects on cell growth; however, EGCG treatment enhanced the expression of ERα, and increased the sensitivity of cells to treatment with an ERα antagonist, tamoxifen. Moreover, EGCG-exposed ERα-negative MDA-MB-231 cell lines, expressing mutated p53, showed a marked decrease in cell growth and up-regulation of ERα and IGF-1R, which resulted in an increased responsiveness of the cell to tamoxifen treatment [251].

There are paradoxical findings on the effect of genistein on the proliferation of ER^+^ and ER^−^ breast cancer cells that are associated with concentration of genistein [252,253]. For instance, ER^+^ and ER^−^ breast cancer cells treated with a high concentration of genistein showed a significant reduction in growth rate, while lower concentrations enhanced their growth rate. Similarly, tamoxifen and SERMs showed controversial effects, which correlated with the applied concentration and the type of tissue [254].

Taken together, the application of phytoestrogens is intricate, due to the controversial effects attributed to variations in doses [255]. Therefore, further comprehensive research is needed to characterize the side effects of using these phytoestrogens, which may be beneficial for endocrine disorder-related public health in the future.

### 7.4. Modulation of the Aromatase Activity

Aromatase, an estrogen synthase, belongs to the cytochrome P450 enzyme family [256,257]. It is highly expressed in breast cancer tissue compared to normal breast tissue [232]. Aromatase inhibitors showed a better capacity for the treatment of breast cancer when compared to tamoxifen [258]. Aromatase stimulation is correlated with ER-independent malignancy [259]. The efficiency of various synthetic aromatase inhibitors in the clinical application of breast cancer treatment, in ER^+^ patients at the postmenopausal stage, was demonstrated [260].

Owing to the similarity between the A and C rings of flavonoids with D and C rings of androstenedione, which is the substrate of aromatase, as well as to the potential of the C4 position’s oxo-group to interact with the heme group of the aromatase, flavonoid compounds potently inhibit aromatase activity [261]. Flavones and isoflavones were reported to bind to estrogen receptors and to the active sites of the aromatase [262]. The potential of flavonoids to influence the promoter activity of aromatase was demonstrated [263,264], additionally their role in the regulation of breast cancer’s aromatase expression has been proven [265].

Aromatase activity is markedly inhibited by luteolin [266], but is up-regulated by hesperetin (3′,5,7-trihydroxy-4-methoxyflavanone) [265]. The imidazolyl quinoline derivative of flavonoids, XHN27, a potent aromatase inhibitor, significantly suppresses the proliferation of breast cancer T47D cells, determined after screening a library of 7000 compounds [267].

### 7.5. Modulation of the Cell Cycle

During carcinogenesis, there is an imbalance between the action of cell cycle progression proteins and cell cycle arrest proteins, resulting in marked cell division and proliferation. Cell cycle progression can be mediated by cyclins and cyclin-dependent kinases (CDK), and its arrest is mediated by CDK inhibitors (CDKi), such as p15, p16, p21, p27, p53, and retinoblastoma tumor suppressor protein (RB). Loss of function of RB, a tumor suppressor gene, is involved in resistance to chemotherapeutic drugs, such as tamoxifen.

Numerous polyphenol-treated cancer cells showed down-regulation of CDK, as well as modulation of CDKi, consequently leading to cell cycle arrest and apoptosis at the G2/M phase [268,269]. In breast cancer cells, the synergy between E2 and IGF-1 is essential for cell cycle progression via up-regulation of Cdk2, Cdk4, and cyclin D1 [270].

In breast and colon cancer cells, ginnalins A–C polyphenols isolated from *Acer saccharum* Marsh. sugar and red maple (*Acer rubrum* L.) species showed remarkable anti-cancer activities via induction of cell cycle arrest, in particular, in the S- and G_2_/M-phases, as well as down-regulation of cyclins A and D1 proteins [268].

The potency of quercetin-3-methyl ether was exploited to induce cell cycle arrest in the G2/M phase, and up-regulation of the phosphorylation level of cyclin B1 (Ser 147) to potently block the growth of breast cancer cells that are resistant or sensitive to lapatinib, a reversible inhibitor of EGFR and HER2 [271]. Therefore, quercetin-3-methyl ether is considered a naturally occurring polyphenol that overcomes the resistance against the common anti-breast-cancer drug, lapatinib. In addition, quercetin-exposed MDA-MB-453 breast cancer cells showed a marked increase in the number of cells in the G2/M phase and a reduction in cell populations in the G1 phase [138].

Quercetin led to down-regulation of cyclin A and cyclin B, and a significant up-regulation of CDK inhibitors, including p53, p21CIP1/waf1, and p27Kip1 [272,273]. As a part of its anti-cancer activities, resveratrol also resulted in the modulation of cell cycle and apoptosis [274].

Curcumin possesses anti-cancer activities via the modulation of apoptosis and the cell cycle [275]. Curcumin-treated human MCF-7 breast cancer cells showed a drastic reduction in proliferation, mediated by cell-cycle arrest in the G_2_/M phase [275]. Curcumin treatment led to apoptotic cell death, which was confirmed by the detection of a high fraction of cells accumulated in the G_0_/G_1_ phase, as well as by the up-regulation of Bax through a p53-dependent mechanism [276]. It was evidenced that curcumin can induce the monopolar spindle formation, accumulation of mitotic arrest deficient 2 (Mad2), and Mad3/BubR1, thereby activating the mitotic checkpoint [277].

Apigenin (4′,5,7-trihydroxyflavone), a flavone, significantly inhibited the proliferation of SK-BR-3 breast cancer cells through inhibition of cell cycle progression at the G2M phase, with the up-regulation of p21^Cip1^, as well as down-regulation of CDK1 and cyclin A and B [278].

EGCG inhibited the division and growth of cancer cells via dephosphorylation of the myosin II regulatory light chain (MRLC), which is essential for contractile ring formation [279]. Consequently, EGCG-treated cells showed high percentages of cell population in the G2/M phase and a decrease in cell growth and division. Of note, EGCG-induced dephosphorylation of MRLC was attributed to its interaction with metastasis-associated 67 kDa laminin receptor (67LR) [279].

### 7.6. Modulation of Apoptosis

Apoptosis is a type of programmed cell death, which is essential for various physiological processes, such as homeostasis and development. Intrinsic or mitochondrial type apoptosis is modulated by the B cell lymphoma (Bcl-2) family proteins [280]. The extrinsic apoptotic pathway is activated by binding of death receptors with their ligands, such as binding of tumor necrosis factor receptor 1 (TNFR1) and tumor necrosis factor (TNF), and the recruitment of receptor-interacting protein (RIP), TNFR1-associated death domain protein (TRADD), and TNFR-associated factor (TRAF), or binding of death-inducing signaling complexes [280].

Apoptosis plays important roles in the potential of quercetin to inhibit the proliferation of human MDA-MB-453 breast cancer cells that are mediated by up-regulation of BAX and down-regulation of Bcl-2 expression, as well as cleavage of caspase-3 and PARP proteins [138]. Quercetin-exposed MCF-7 breast cancer cells showed apoptotic cell death with a reduction in mitochondrial membrane potential, down-regulation of Bcl-2 protein, and activation of the initiator caspases, caspase-8 and caspase-9, and the effector caspase, caspase-6, which were attributed to the binding of quercetin to the Fas/CD95 receptor [273]. Moreover, quercetin significantly inhibited MD-MBA-231 breast cancer cells through the activation of caspase-3/-8/-9 [281].

Apigenin-treated SK-BR-3 breast cancer cells showed apoptotic cell death, evidenced by the up-regulation of p53 and its downstream effectors, BAX and cytochrome c [278]. A recent study detected a dramatic decrease in cell proliferation, as well as significant stimulation of apoptosis signaling pathways, such as PARP cleavage and caspase-8 and -9 cleavages in apigenin-treated SKBR3 breast cancer cells [282]. This study concluded that STAT3 inhibition mediated apigenin-enhanced apoptosis signaling pathways in SKBR3 cells.

On treatment with green tea polyphenols and EGCG, a significant reduction in cell growth associated with apoptotic changes, such as stimulation of BAX, cleavage of PARP, and down-regulation of Bcl-2, was observed in MD-MB-231 human breast cancer cells [283].

Resveratrol treatment led to apoptotic cell death in T47D breast cancer cells via activation of CD95L, which is involved in the extrinsic apoptotic pathway [284], as well as activation of p53 [285]. PARP cleavage was significantly induced in resveratrol-treated MDA-MB-231 cells, and was correlated with the activation of caspase-3 [286]. Moreover, resveratrol induced apoptosis in various malignant cells (including MDA-MB-231 and MDA-MB-468 cell lines), via inhibition of Src tyrosine kinase activity and blockage of STAT3 activation [287]. In estrogen-positive breast cancer cells, resveratrol markedly reduced growth rate by stimulating apoptosis through reduction of the ratio of Bcl2/BAX, which was independent of the presence of E2 [288]. Therefore, resveratrol is considered a potential and safe chemopreventive alternative to hormone replacement therapy (HRT), in particular, in postmenopausal women, and against hormone-dependent breast cancer.

Genistein-exposed MCF-7 cells showed up-regulation of BAX and reduction of Bcl-2 at the protein and mRNA levels, resulting in a reduction in the Bcl-2/BAX ratio [289]. This effect is mediated by blocking the activation of the IGF receptor (IGFR), as well as the phosphorylation of AKT.

Fisetin (3,3′,4′,7-tetrahydroxyflavone), a flavonoid, which is widely distributed in fruits and vegetables, induced an uncommon form of apoptosis in caspase-voided MCF-7 cells characterized by the activation of caspase-7/-8/-9, cleaved PARP, mitochondrial membrane depolarization, up-regulation of p53, and break in the plasma membrane, while no change was detected in DNA or phosphatidylserine (PS) [290]. These apoptotic changes were abolished upon treatment with a pan-caspase inhibitor, z-VAD-fmk.

### 7.7. Modulation of the Multidrug Resistance (MDR)

Despite the potency of anti-cancer drugs in decreasing cancer size, a few populations of CSCs potently resist chemotherapy and lead to tumor recurrence and MDR [291]. The crosslink between the virulence of CSCs and MDR is correlated with reduction of intracellular concentrations of anti-cancer drugs, continual growth, and cancer relapse [292].

The emergence of MDR is linked to over-expression of the ATP-binding cassette (ABC) transporters family, which is composed of energy-dependent transporter proteins, which act as pumps. ABC transporters are involved in drug efflux, thereby decreasing in intracellular concentrations [293]. Transporter proteins include various proteins, such as multidrug resistant-associated proteins (MRPs), mitoxantrone resistance protein (MXR or ABCG2), and P-glycoprotein (P-gp) or ABCB1.

EGCG treatment leads to the accumulation of rhodamine-123 dye in MDR cell lines and an increase in the intracellular concentration of anti-cancer drugs [294]. Moreover, a group of six common polyphenols (naringenin, silymarin, daidzein, quercetin, resveratrol, and hesperetin) potently inhibit the activity of MRP family proteins, thereby inhibiting efflux [295].

Curcumin treatment leads to down-regulation of MDR-1b expression by its interaction with PI3K/AKT/NF-κB signaling [296]. Moreover, it enhances the sensitivity of MDR cell lines to chemotherapeutic agents, such as cisplatin, vincristine, doxorubicin, tamoxifen, and mitoxantrone [297].

### 7.8. Modulation of Signaling Pathways Related to Self-Renewal Capacity and Transformation of CSCs

CD44^+^/CD24^low^ BCSCs showed a high degree of tumorigenicity with enhanced sphere formation and self-renewal capacities [298,299]. Embryonic development-related signaling pathways, such as Notch, Wnt/β-catenin, and Hedgehog, were significantly implicated in the self-renewal property of BCSCs [300]. We will discuss the potential of polyphenols to interfere with the stemness-related signaling pathways below.

#### 7.8.1. Hedgehog (Hh) Signaling Pathway

Hh, encoding secreted proteins, modulates cellular differentiation, proliferation, and development processes via autocrine- and paracrine-mediated signaling pathways [301]. There are three main mammalian homologs of the Hh gene, namely Sonic hedgehog (Shh), Indian hedgehog, and Desert hedgehog [302]. The interaction of the Hh proteins with the transmembrane protein, patched (PTC), leads to activation or phosphorylation of another transmembrane protein, smoothened (SMO) [303]. The Hh pathway is correlated with the development and maintenance of CSCs in breast cancer, myeloid leukaemia, glioma, gastric cancer, and multiple myeloma [304,305,306,307]. Therefore, the discovery of new inhibitors targeting the Hh signaling pathway is a potent anti-cancer strategy and is under clinical trials (phases I and II) [308].

Cyclopamine, extracted from *Veratrum californicum* or corn lily, was the first discovered phytochemical that inhibits Hh signaling pathways by inactivation of SMO [306,309]. Cyclopamine inhibits breast CSC proliferation and mammosphere formation [304].

Genistein potently inhibits the growth of CD44^+^/CD24^−^BCSCs by the notable down-regulation of mRNA levels and the protein levels of SMO and Gli1, which are key factors for modulation of Hedgehog-Gli1 signaling [310].

#### 7.8.2. Notch Signaling Pathway

Notch proteins are composed of four transmembrane glycoproteins, namely, Notch1, Notch2, Notch3, and Notch4, and also have five ligands, Delta-like1, Delta-like3, Delta-like4, Jagged1, and Jagged2 [311]. The Notch signaling pathway is involved in cellular proliferation and differentiation [312]. Its activation is mediated by the interaction between the extracellular domains of receptors with ligands and the release of the Notch intracellular domain (NICD) into the nucleus through proteolytic cleavage.

Resveratrol leads to down-regulation of Notch proteins only at the post-translational level, a decrease in mRNA levels of pre-TCRα and HES1, an increase in p53, and a reduction of PI3K/AKT signaling in MOLT-4 acute lymphoblastic leukemia cells [313].

#### 7.8.3. Wingless/Integration 1 (Wnt) and the β-Catenin Signaling Pathway

The Wnt/β-Catenin signaling pathway is considered one of the essential signaling pathways for the self-renewal of BCSCs [314]. β-Catenin is an integral effector of the Wnt signaling pathway in the nucleus. In response to Wnt activation, stabilized β-catenin moves to the nucleus and activates target genes by its interaction with the TC/LEF transcription factor [314,315]. Glycogen synthase kinase3β (GSK3β), axin, casein kinase1α, and adenomatous polyposis coli (APC) protein complex are linked to regulation of the intracellular level of β-Catenin.

EGCG significantly inhibits the formation and invasiveness of breast cancer by suppressing the Wnt signaling pathway and reducing c-myc expression [316]; additionally, it potently reduces nuclear β-Catenin [317]. Curcumin also targets β-Catenin in the caspase-mediated mechanism in colon cancer [318]. Sulforaphane, a product of the conversion of glucoraphanin, which is the main glucosinolate in broccoli and its sprouts, has potent chemoprevention activity against a wide range of cancers [319,320,321].

Sulforaphane-exposed human cervical carcinoma and hepatocarcinoma cell lines show a significant increase in apoptosis by degradation of the β-Catenin protein [322]. Sulforaphane potently eliminates BCSCs in vitro and in vivo by targeting the Wnt/β-Catenin-mediated self-renewal property of BCSCs [321].

Piperine, an alkaloid isolated from black pepper (*Piper nigrum*) and long pepper (*Piper longum*), shows potent in vivo reduction of lung metastasis [323]; in addition, it inhibits the self-renewal property of BCSCs through down-regulation of the Wnt signaling pathway [324].

Oxymatrine, an alkaloid isolated from *Sophora japonica*, markedly decreases the proliferation of breast cancer and its drastic reduction of the growth of the sorted side population (SP) of CSCs was demonstrated [325]. In addition, it significantly reduced the activity of the Wnt/β-catenin signaling pathway.

### 7.9. Modulation of Autophagy

Autophagy plays a pivotal role in maintaining stem cell characteristics. Conditional deletion of Atg7 leads to a loss in properties, and disturbance in hematopoietic stem cell function [326]. In BCSCs, a high basal level of autophagy was detected in ALDH1+ cell populations [327]. Autophagy is essential for the enhancement of the invasiveness and metastatic properties of glioblastoma stem cells, which are mediated by DRAM1 and p62 [328].

Rottlerin significantly inhibits the growth of human BCSCs and induces autophagy via up-regulation of Atg12 and Beclin-1, and conversion of LC3-I into LC3-II [329]. Up-regulation of BAX, reduction in phosphorylation of AKT, mTOR, and AMPK, and a significant decrease in the expression of anti-apoptotic factors, were demonstrated over a long period of time of rottlerin treatment. shRNAs targeting Atg7 and Beclin-1 abrogated the capacity of rottlerin to induce autophagy. Autophagy inhibitors, 3-MA, Baf-A1, and cycloheximide, alleviate rottlerin-induced apoptosis and phosphorylation of AMPK. Inactivation of AMPK was concomitant with the down-regulation of Beclin-1, Atg12, and LC3.

Resveratrol blocks the growth of BCSCs and number of mammospheres [330]. It showed significant up-regulation of LC3-II, Atg7, and Beclin1, which is concomitant with cell toxicity.

### 7.10. Modulation of the Epithelial Mesenchymal Transition (EMT)

EMT is an intricate developmental process, in which special differentiated polarized epithelial cells undergo morphogenesis via loss of their differentiation characteristics, such as cell–cell adhesion, cell polarity, immotile status, and the transformation into mesenchymal cells with invasive and migratory properties [331,332].

During EMT, there is a decrease in the expression of epithelial markers, such as γ-catenin and E-cadherin, and up-regulation of mesenchymal markers, including vimentin, N-cadherin, fibronectin, and MMP-2/9. In contrast, mesenchymal-epithelial transition (MET) takes place after the migration and invasion of cells to their designated sites [333]. E-cadherin, encoded by CDH1, plays a pivotal role in the inhibition of tumor invasiveness and malignancy, as well as suppression of EMT.

There are various transcription factors, the Snail superfamily of zinc-finger transcriptional repressors, such as Snail 1 and Snail 2 (also known as slug); the ZEB family, such as ZEB1 (also known as TCF8 and δEF1) and ZEB2 (also known as Smad-interacting protein 1 (SIP1); and basic helix-loop-helix (bHLH), such as E47 (also known as TCF3), TCF4 (also known as E2-2), and TWIST1 [331], which represent transcription repressors of the CDH1 gene, and, thereby, inhibit tumor malignancy and invasiveness.

Up-regulation of the EMT transcription repressor is induced by a complicated signaling network that is enhanced by receptor tyrosine kinases (RTKs) and transforming growth factor β (TGFβ) [334]. The NF-κB signaling pathway is involved in the activation of Snail, Slug, ZEB-1/2, and Twist, as well as the up-regulation of mesenchymal markers, including MMPs, fibronectin, and vimentin [335].

Resveratrol treatment triggers apoptosis and recovers the expression of γ-catenin and E-cadherin in tamoxifen-resistant breast cancer cells (MCF-7/TR) via targeting of TGFβ and its downstream effector, Smad [336]. Moreover, resveratrol recovered epithelial characteristics in EGF-transformed breast cancer cell lines via repression of the ERK1/2 signaling pathways [337].

Baicalin and baicalein, the main flavones isolated from *Scutellaria baicalensis*, potently inhibit EMT by targeting TGF-β1, and the inactivation of NF-κB-induced activation of Slug [338]. Furthermore, Chrysin (5,7-dihydroxyflavone), a flavone isolated from passion flower (*Passiflora caerulea*) and honeycomb of *Apis mellifera*, significantly inhibits the metastatic and invasive characteristics of TNBC through down-regulation of Slug, Snail, and Vimentin, and inhibition of MMP-10 via blocking of the PI3K-AKT signaling pathway [339].

Honokiol, isolated from seed cones from *Magnolia grandiflora* led to inhibition of EMT, which was mediated by inactivation of STAT3 and, in turn, blocked off the repressive action of ZEB1 on E-cadherin [340].

To sum up, several interesting studies have showed the capacity of polyphenols to potently restore epithelial characteristics in transformed breast cancer cells, and prevented the emergence of CSC phenotype and drug resistance.

## 8. Conclusions and Perspectives

In this review, we provide detailed information on the broad spectrum of mechanistic actions of polyphenols against breast cancer and CSCs. Many studies revealed that apoptosis- and/or autophagy-related signaling pathways are modulated by polyphenol treatment. Pharmacological inhibition of autophagy plays a pivotal role in polyphenol-induced cell death. We also explained the potential of polyphenols to target breast cancer and CSCs via modulation of various stemness-related signaling pathways and transcription factors.

This review provides useful information that will guide future research, which will provide strategies for efficient, polyphenol-based prevention, or treatment, of breast cancer. Further efforts are needed to resolve several remaining hurdles, such as the variations in applied dose, the large discrepancy between the in vitro and in vivo doses, and exposure time. Moreover, a better understanding of the interconnection between apoptosis and autophagy in the polyphenol-mediated treatment of breast cancer is needed to characterize the key factors involved in the actions of polyphenols. However, the progress in technology continues to provide answers to unresolved questions. To determine the potential of polyphenols in curing breast cancer in clinical trials, discovered polyphenols need to be elucidated. Chemotherapy remains.

In fact, there is a paucity of information related to the application of polyphenols as chemopreventive compounds. What lies ahead is the application of previously-discovered polyphenols in the treatment of breast cancer in clinical trials. Collectively, the therapeutic applications of polyphenols in breast cancer are promising, as these compounds present various mechanistic actions and their clinical applications need to be tested.
